# Comparisons of Neuronal and Excitatory Network Properties between the Rat Brainstem Nuclei that Participate in Vertical and Horizontal Gaze Holding

**DOI:** 10.1523/ENEURO.0180-17.2017

**Published:** 2017-09-13

**Authors:** Yasuhiko Saito, Taketoshi Sugimura, Yuchio Yanagawa

**Affiliations:** 1 Department of Neurophysiology, Nara Medical University, Kashihara, Nara 634-8521, Japan; 2 Department of Genetic and Behavioral Neuroscience, Gunma University Graduate School of Medicine, Maebashi, Gunma 371-8511, Japan; 3 Japan Science and Technology Agency, Core Research for Evolutional Science and Technology (CREST), Sanbancho, Chiyoda-ku, Tokyo 102-0075, Japan

**Keywords:** Ca2+-permeable AMPA receptor, firing pattern, interstitial nucleus of Cajal, local excitatory network, neural integrator, prepositus hypoglossi nucleus

## Abstract

Gaze holding is primarily controlled by neural structures including the prepositus hypoglossi nucleus (PHN) for horizontal gaze and the interstitial nucleus of Cajal (INC) for vertical and torsional gaze. In contrast to the accumulating findings of the PHN, there is no report regarding the membrane properties of INC neurons or the local networks in the INC. In this study, to verify whether the neural structure of the INC is similar to that of the PHN, we investigated the neuronal and network properties of the INC using whole-cell recordings in rat brainstem slices. Three types of afterhyperpolarization (AHP) profiles and five firing patterns observed in PHN neurons were also observed in INC neurons. However, the overall distributions based on the AHP profile and the firing patterns of INC neurons were different from those of PHN neurons. The application of burst stimulation to a nearby site of a recorded INC neuron induced an increase in the frequency of spontaneous EPSCs. The duration of the increased EPSC frequency of INC neurons was not significantly different from that of PHN neurons. The percent of duration reduction induced by a Ca^2+^-permeable AMPA (CP-AMPA) receptor antagonist was significantly smaller in the INC than in the PHN. These findings suggest that local excitatory networks that activate sustained EPSC responses also exist in the INC, but their activation mechanisms including the contribution of CP-AMPA receptors differ between the INC and the PHN.

## Visual Abstract

**Figure:**
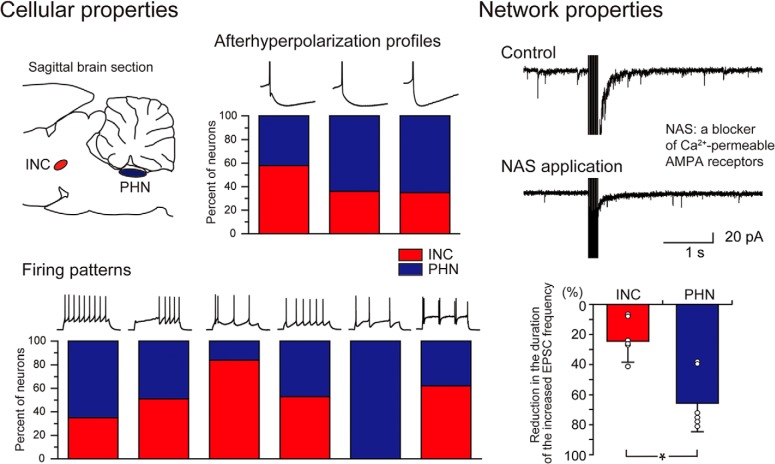

## Significance Statement

Gaze holding is controlled primarily by neural structures including the prepositus hypoglossi nucleus (PHN) for horizontal gaze and the interstitial nucleus of Cajal (INC) for vertical gaze. In this study, to verify whether the neural structure of the INC is similar to that of the PHN, we compared the neuronal and network properties between the INC and PHN. The overall distributions of INC neurons based on their electrophysiological properties were different from those of PHN neurons. The local excitatory networks that activated the sustained EPSC responses were present in both the INC and PHN, but their activation mechanisms including the contribution of Ca^2+^-permeable AMPA (CP-AMPA) receptors differed between the INC and PHN.

## Introduction

Holding the eye steady after movement is essential for stably capturing a visual target by the foveal centralis of the retina. Gaze holding is generated primarily by neural structures that function as an oculomotor neural integrator, transforming transient burst signals that are proportional to eye or head velocity into sustained signals that are proportional to eye position (Robinson, 1975, 1989; Fukushima et al., 1992; Fukushima and Kaneko 1995; Moschovakis 1997; Leigh and Zee, 2015). In the oculomotor system, horizontal and vertical (and torsional) eye movements are controlled separately by different brain areas such as the paramedian pontine reticular formation and the rostral interstitial nucleus of the medial longitudinal fasciculus, respectively (Miller, 1985; Büttner and Büttner-Ennever, 2006). Similar to the eye movement system, oculomotor neural integrators are separated into the prepositus hypoglossi nucleus (PHN) for horizontal gaze and the interstitial nucleus of Cajal (INC) for vertical gaze (Fukushima, 1987, 1991; Fukushima et al., 1992; Fukushima and Kaneko, 1995; Moschovakis, 1997; Leigh and Zee, 2015). The PHN is located in the pontomedullary region and contains neurons of various sizes (McCrea and Horn, 2006), whereas the INC is located in the rostral mesencephalon and consists of scattered neurons (Rutherford and Gwyn, 1982; Fukushima, 1987). Both nuclei contain a variety of neurons that encode signals related to eye movements, including eye position (PHN, Lopez-Barneo et al., 1982; Delgado-García et al., 1989; Escudero et al., 1992; McFarland and Fuchs, 1992; INC, King et al., 1981; Fukushima et al., 1990a, b; Crawford et al., 1991; Helmchen et al., 1998; Chimoto et al., 1999). Although several models have been proposed regarding the neural mechanisms of signal transformation in the neural integrator (Cannon et al., 1983; Galiana and Outerbridge, 1984; Cannon and Robinson, 1985; Arnold and Robinson, 1997; Draye et al., 1997; Seung et al., 2000; Major and Tank, 2004), the detailed mechanisms have not been elucidated experimentally, except for findings reported in a series of studies in goldfish (Aksay et al., 2001, 2003, 2007; Lee et al., 2015).

Apart from the findings of neurons that show spiking in relation to eye movements, intracellular recordings using *in vitro* slice preparations have demonstrated the presence of a variety of neurons that exhibit distinct electrophysiological membrane properties in the PHN (guinea pig, Bobker and Williams, 1990, 1995; Idoux et al., 2006, 2008; mouse, Kolkman et al., 2011; rat, Shino et al., 2008, 2011; Saito et al., 2012, 2015; Saito and Yanagawa, 2013; Zhang et al., 2014). Idoux et al. (2006) found a unique neuron type (type D) that exhibited clusters of action potentials intermingled with subthreshold membrane oscillations and bistable plateau-like responses through an NMDA-dependent mechanism, which may be used for the robustness of neural activities in the integrator network. Furthermore, a previous study demonstrated the presence of local excitatory networks in the PHN (Saito and Yanagawa, 2010). These networks were activated via Ca^2+^-permeable AMPA (CP-AMPA) receptors in addition to NMDA receptors and participated in sustained activity that may code eye position. In contrast to the accumulating findings of the neuronal and network properties in the PHN, there is no report regarding the membrane properties of INC neurons and the local networks in the INC. The comparison of the neuronal and network properties between the PHN and the INC can provide information regarding whether the mechanisms of the neural integrators are common or different in the horizontal and vertical systems. In the present study, to clarify whether the neural structure of the INC is similar to that of the PHN, we investigated the neuronal and network properties of the INC using whole-cell recordings in rat brainstem slices. Furthermore, we compared the results obtained from the INC to those obtained from previous studies of the PHN (Shino et al., 2008; Saito and Yanagawa, 2010; Zhang et al., 2014; Saito et al., 2015) to verify (1) whether the neuronal distributions classified by membrane properties in the INC are similar to those in the PHN and (2) whether the local excitatory networks that induce the sustained activity are also present in the INC.

## Materials and Methods

All experimental procedures were approved by the Animal Care and Experimentation Committee of Gunma University and the Animal Care Committee of Nara Medical University, and the experiments were conducted in accordance with the Guidelines outlined by the United States National Institutes of Health regarding the care and use of animals for experimental research. Every effort was made to minimize suffering and the number of animals used in these experiments. The data of INC neurons and networks were newly obtained and analyzed in this study; however, the results of the PHN were reanalyzed using data obtained in our previous studies (Shino et al., 2008; Saito and Yanagawa, 2010; Saito et al., 2015).

### Animals and histologic procedures

The data were obtained from wild-type Wistar rats and vesicular GABA transporter (VGAT)-Venus transgenic rats (Uematsu et al., 2008; Shino et al., 2011). In VGAT-Venus rats, inhibitory neurons that expressed a fluorescent protein Venus (Nagai et al., 2002) were easily identifiable with fluorescence microscopy. A total of 18 young wild-type and 11 VGAT-Venus rats [17–21 postnatal days (PND)] of either sex were used in the present study. In addition, two double transgenic rats were used for histologic observation of inhibitory and cholinergic neurons in the INC and PHN. The double transgenic rats were generated by crossing the choline acetyltransferase (ChAT)-tdTomato and VGAT-Venus transgenic rats (Saito et al., 2015; Zhang et al., 2016). These rats (21 PND) were transcardially perfused with 0.05 M phosphate buffer (PB, pH 7.4), followed by 4% paraformaldehyde in PB under deep anesthesia induced via isoflurane inhalation (vaporized with oxygen) followed by intraperitoneal injection of sodium pentobarbital (>50 mg/kg). The brain areas including the PHN and INC were dissected and cut frontally into 50-µm sections using a Microslicer (Dosaka EM). The sections were mounted on MAS-coated slides (Matsunami Glass) using an anti-fade medium (ProLong Gold anti-fade reagent, Invitrogen). The neurons were observed under a fluorescence microscope (BX60, Olympus). In this preparation, inhibitory and cholinergic neurons express yellow-green and red fluorescence, respectively.

### Slice preparation and whole-cell recording

Wild-type or VGAT-Venus rats were decapitated under deep anesthesia with isoflurane, and the brain was quickly removed. Frontal slices (250 or 400 μm thick), including the INC were sliced using a Microslicer (Pro 7, Dosaka EM). After the slices were incubated for >1 h at room temperature in an extracellular solution containing 125 mM NaCl, 2.5 mM KCl, 2 mM CaCl_2_, 1 mM MgCl_2_, 1.25 mM NaH_2_PO_4_, 26 mM NaHCO_3_, and 25 mM glucose and aerated with 95% O_2_ and 5% CO_2_ (pH 7.4), they were transferred to a submerged recording chamber on an upright microscope (Leica DM LFS, Leica) and continuously perfused with extracellular solution at a rate of 5 ml/min. The bath temperature was kept at 30–32°C using an in-line heater (SH-27A, Warner Instruments). Whole-cell current-clamp and voltage-clamp recordings were performed using an EPC-8 patch clamp amplifier (HEKA). Patch pipettes were prepared from borosilicate glass capillaries and filled with a K^+^-based internal solution containing 120 mM K-methylsulfate, 10 mM KCl, 0.2 mM EGTA, 2 mM Mg-ATP, 0.3 mM Na-GTP, 10 mM HEPES, 10 mM Na_2_-phosphocreatine, and 0.1 mM spermine, pH adjusted to 7.3 with KOH for current-clamp experiments. The INC was identified by referring to a rat brain atlas (Paxinos and Watson, 2007). Although the medial boundary of the INC was easily defined by the medial longitudinal fasciculus, the lateral boundary of the INC was not as clear. Therefore, we did not attempt to record from the neurons that located near the lateral boundary. The range between −6.12 and −5.04 mm from the bregma shown in the atlas corresponded to the range of slices used in this study. However, the rats used in this study were younger than those used in the atlas, and thus, we used two to three slices (250 μm thick) or one to two slices (400 μm thick) per animal. In voltage-clamp experiments, we used a Cs^+^-based internal solution containing 145 mM Cs-gluconate, 5 mM CsCl, 0.2 mM EGTA, 2 mM Mg-ATP, 0.3 mM Na-GTP, 10 mM HEPES, 0.1 mM spermine, and 5 mM lidocaine *N*-ethyl bromide (QX-314), pH 7.3. The osmolarity of the internal solution was 280–290 mOsm/l and the resistance of patch electrodes was 5–8 MΩ in the bath solution. Voltage and current signals were low-pass filtered at 3 kHz and digitized at 10 kHz. The measured liquid junction potential (K^+^-based internal solution = −5 mV, Cs^+^-based internal solution = −10 mV) was corrected. Neurons displaying a membrane potential below −50 mV immediately after patch membrane rupture and action potential peaks higher than 0 mV were used for further analyses. When voltage responses to current pulses were investigated in current-clamp mode, depolarizing current pulses (up to 400 pA in 20- to 40-pA increments, 400 ms in duration) were injected into the neurons. In these neurons, the membrane potential was maintained at −85 to −75 mV before the current pulses via the injection of constant currents. For analyses of the action potential profiles, including the afterhyperpolarization (AHP) profiles, the depolarizing current pulses were adjusted to induce one action potential over 400 ms. When current recordings were performed in voltage-clamp mode, the holding potential was set to −75 mV. For the application of the high frequency stimulation (burst stimulation), electrical stimulation of 20 cathodal square-wave pulses (10–25 μA, 100 μs in duration) was applied in the vicinity of a recorded neuron using a glass micropipette that was filled with extracellular solution. To determine the stimulation site, we scanned 8–10 different sites around a recorded neuron with the application of burst stimulation to each site. A site where the current responses of the neuron appeared to be the largest was determined as an appropriate stimulation site. The approximate distance between each recorded neuron and the stimulation electrode was 30–80 μm. The burst stimulation was applied to INC neurons in which spontaneous EPSCs occurred at a frequency of >1 event/s. The data were acquired using a pClamp9 system (Molecular Devices). Strychnine hydrochloride and 1-naphthyl acetyl spermine (NAS) was purchased from Sigma-Aldrich Japan, and other drugs including pictrotoxin were purchased from Wako Pure Chemical Industries.

### Data analysis

**Figure 1.**
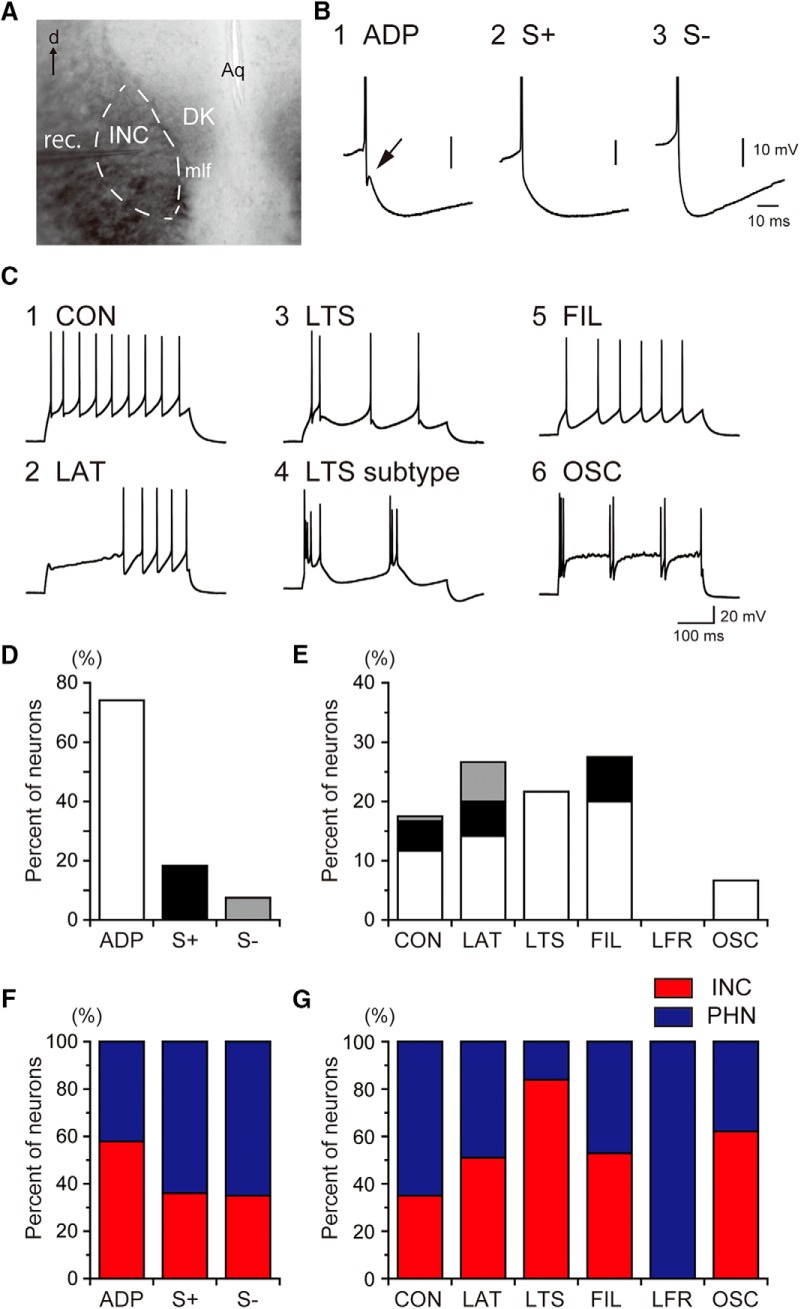
AHP profiles and firing patterns of INC neurons. ***A***, Photomicrograph of a frontal section of the INC. The dashed line shows the rough boundary of the INC. DK, nucleus of Darkschewitsch; mlf, medial longitudinal fasciculus; Aq, aqueduct; rec, recording pipette; d, dorsal. ***B***, AHP profiles. (1) AHP with ADP (arrow), (2) AHP with a slow component (S+), and (3) AHP without a slow component (S-). C, firing patterns. (1) Continuous spiking pattern (CON), (2) late spiking pattern (LAT), (3) low-threshold calcium spike pattern (LTS), (4) a subtype of LTS that exhibited oscillatory burst firing, (5) longer first interspike interval pattern (FIL), and (6) oscillatory firing pattern (OSC). The white, black and gray bars represent the ADP, S+ and S- profiles, respectively. ***D***, ***E***, distributions of the AHP profiles (***D***) and the firing patterns (***E***) among INC neurons. ***F***, ***G***, The proportion of INC (red) and PHN (blue) neurons that exhibited each AHP profile (***F***) or firing pattern (***G***). The percentages of neurons that exhibited each AHP profile or each firing pattern was normalized to the total percentages of neurons exhibiting each property.

Off-line analysis was performed with AxoGraph X software (RRID: SCR_014284) and the Kaleidagraph (RRID: SCR_014980). The input resistance was estimated based on the voltage change induced by a hyperpolarizing current pulse of −40 pA applied at a membrane potential of −55 to −65 mV. The amplitude of the AHP was estimated as the difference between the most negative membrane potential of the AHP and the action potential threshold, which was defined as the membrane potential at which the derivative of the voltage trace reached 10 V/s. To clarify which neurons were predominant in each AHP profile or each firing pattern between the INC and the PHN, we presented the proportion of INC and PHN neurons that exhibited each AHP profile or each firing pattern (see Figures 1, *F* and *G*, and 3, *C* and *D*). The total number of INC and PHN neurons was each normalized to be 100, and the percentages of neurons that exhibited each AHP profile or each firing pattern were obtained by dividing the number of either INC or PHN neurons by the sum of the number of INC or PHN neurons, respectively, exhibiting each property. EPSCs were determined when the peak of the inward current was more than three times the SD of the baseline noise before burst stimulation. To detect EPSCs after burst stimulation, an inward deflection of the baseline, which occurred immediately after burst stimulation and returned to the origin by ∼300 ms (see Figure 4*A1* below), was removed by the subtraction of the trace filtered up to 10 Hz from the original trace. The EPSC frequencies before and after burst stimulation were measured from recordings of 2 s before and 1 s after burst stimulation, respectively. To estimate the duration of the increased EPSC frequency, we generated a histogram of EPSC frequency versus time (see Figure 4*A* below). Using the histogram, the duration was defined as the time period from when burst stimulation was terminated to when the averaged value of three adjacent bins (corresponding to 300 ms) became equal to or smaller than the average baseline EPSC frequency before burst stimulation. When the effect of NAS on the EPSC responses was investigated, we measured the amplitude of EPSC and the duration of the increased EPSC frequency. Because the application of NAS usually reduced the baseline noise, low amplitude EPSCs were detected in the presence of NAS. Therefore, a simple comparison of the average EPSCs in the presence of NAS to those in the control may lead to overestimation. Instead, we focused on the maximum EPSC before burst stimulation and estimated the reduction in the amplitude of the maximum EPSC by the application of NAS. The maximum EPSC of the recorded neuron was obtained by averaging the maximum EPSC in each of five recordings. All values are shown as the mean ± SD, and the error bars in the figures represent the SD. The number (*n*) refers to the number of neurons analyzed unless otherwise noted. The statistical analysis was performed using unpaired or paired Student’s *t* tests for normally distributed data and Mann-Whitney tests for the data that did not follow a normal distribution. Data normality was determined using the Shapiro-Wilk test. StatView software (Hulinks) and JMP software (RRID: SCR_014242) were used for the analyses. A *post hoc* power analysis was performed using G*Power3 software (http://www.gpower.hhu.de/; RRID: SCR_013726; Faul et al., 2007). Statistical significance was determined at the level of *p* < 0.05. Detailed results of the statistical analyses are shown in Table 1.

**Table 1.** Statistical test

Panel, parameter (bivariate)	Distribution and *p* value (type of test)	Power (α = 0.05)
Figure 3*E2*, frequency of SMOCs (INC vs PHN)	Non-normal 0.081 (Mann-Whitney test)	0.389
Figure 4*B*, duration (INC vs PHN)	Normal 0.053 (unpaired *t* test)	0.627
Figure 4*C*, F_post_/F_pre_ (INC vs PHN)	Non-normal 0.016 (Mann-Whitney test)	0.801
Figure 4D2, max. EPSC amplitude (control vs NAS)	Normal 0.0005 (paired *t* test)	1.000
Figure 4D*3*, duration (control vs NAS)	Normal 0.011 (paired *t* test)	0.937
Figure 4*E*, reduction in max. EPSC amp. (INC vs PHN)	Non-normal 0.098 (Mann-Whitney test)	0.520
Figure 4*F*, reduction in duration (INC vs PHN)	Non-normal 0.0087 (Mann-Whitney test)	0.997
Table 2, input capacitance (V+ INC vs V+ PHN)	Non-normal <0.0001 (Mann-Whitney test)	1.000
Table 2, input resistance (V+ INC vs V+ PHN)	Non-normal <0.0001 (Mann-Whitney test)	1.000
Table 2, spike amplitude (V+ INC vs V+ PHN)	Non-normal 0.106 (Mann-Whitney test)	0.517
Table 2, spike half-width (V+ INC vs V+ PHN)	Non-normal 0.0097(Mann-Whitney test)	0.689
Table 2, AHP amplitude (V+ INC vs V+ PHN)	Non-normal 0.0007 (Mann-Whitney test)	0.950
Table 2, spontaneous firing rate (V+ INC vs V+ PHN)	Non-normal 0. 559 (Mann-Whitney test)	0.305
Table 2, CV of interspike interval (V+ INC vs V+ PHN)	Non-normal 0.036 (Mann-Whitney test)	0.668
Table 2, input capacitance (V- INC vs D- PHN)	Non-normal 0.027 (Mann-Whitney test)	0.380
Table 2, input resistance (V- INC vs D- PHN)	Non-normal 0.0002 (Mann-Whitney test)	0.991
Table 2, spike amplitude (V- INC vs D- PHN)	Normal <0.0001 (unpaired *t* test)	0.989
Table 2, spike half-width (V- INC vs D- PHN)	Non-normal <0.0001 (Mann-Whitney test)	0.961
Table 2, AHP amplitude (V- INC vs D- PHN)	Non-normal 0.0063 (Mann-Whitney test)	0.872
Table 2, spontaneous firing rate (V- INC vs D- PHN)	Non-normal 0.108 (Mann-Whitney test)	0.284
Table 2, CV of the interspike interval (V- INC vs D- PHN)	Non-normal 0.0003 (Mann-Whitney test)	0.786

## Results

### AHP profiles and firing patterns of INC neurons

In previous studies of PHN neurons (Shino et al., 2008, 2011; Zhang et al., 2014; Saito et al., 2015), AHP profiles and firing patterns, which are the parameters that determine the firing behavior and the output properties of neurons, respectively, were characterized. In PHN neurons, AHP profiles and firing patterns were classified into three and six types, respectively. The three AHP profiles included (1) an AHP that contained a slow component and afterdepolarization (ADP), (2) an AHP that contained a slow component (S+), and (3) an AHP that lacked a slow component (S-). The six firing patterns included (1) a repetitive firing pattern with relatively constant interspike intervals (CON), (2) a firing pattern with a delay in the generation of the first spike because of transient hyperpolarization following the onset of the depolarizing pulse (LAT), (3) a firing pattern that exhibited a cluster of two or more spikes because of a low-threshold calcium spike (LTS), (4) a firing pattern in which the first interspike interval was longer compared with the second interval (FIL), (5) a firing pattern that exhibited few spikes during the 400-ms current injection, despite sufficient membrane depolarization (LFR), and (6) a firing pattern that exhibited an oscillatory property (OSC). Therefore, we investigated whether these AHP profiles and firing patterns are also applicable to INC neurons and which AHP profiles and firing patterns were preferentially found in INC neurons. When we performed whole-cell recordings from randomly sampled neurons (*n* = 120) in the INC (Fig. 1*A*), the three types of the AHP profile were observed in the neurons (Fig. 1*B*). The distribution of INC neurons classified by AHP profiles revealed that >70% of recorded INC neurons exhibited the ADP profile (Fig. 1*D*). When the firing patterns were analyzed, five firing patterns, namely, the CON, LAT, LTS, FIL, and OSC patterns, were observed (Fig. 1*C*). Interestingly, three neurons showed repeated burst firing during the application of current pulses (Fig. 1*C4*), which had not been observed in PHN neurons. However, all the neurons with this firing pattern exhibited transient burst firing that was caused by LTS above the threshold of spike generation. We checked the position of these neurons after the recordings and confirmed that all these neurons were definitely within the INC. Therefore, these neurons were regarded as a subtype of the LTS type. INC neurons that exhibited the LFR pattern were not observed in this study. Figure 1*E* shows the distribution of INC neurons classified based on firing patterns. Except for the OSC type, which represented a minor population, no preferential firing type was apparent in INC neurons. To clarify which neurons were predominant in each AHP profile or each firing pattern between the INC and the PHN, we compared the percentage of INC neurons to that of PHN neurons obtained from a previous study (Shino et al., 2008). The percentage of neurons exhibiting the ADP profile was higher in the INC (red) than in the PHN (blue), whereas the percentages of neurons exhibiting the other two profiles were higher in the PHN than in the INC (Fig. 1*F*). Regarding the distribution of neurons classified based on the firing patterns (Fig. 1*G*), a higher percentage of PHN neurons exhibited the CON pattern, and a higher percentage of INC neurons exhibited the LTS pattern. As described above, no neuron exhibited the LFR pattern in the INC, although ∼10% of PHN neurons exhibited this pattern.

### AHP profiles and firing patterns of excitatory and inhibitory neurons

**Figure 2.**
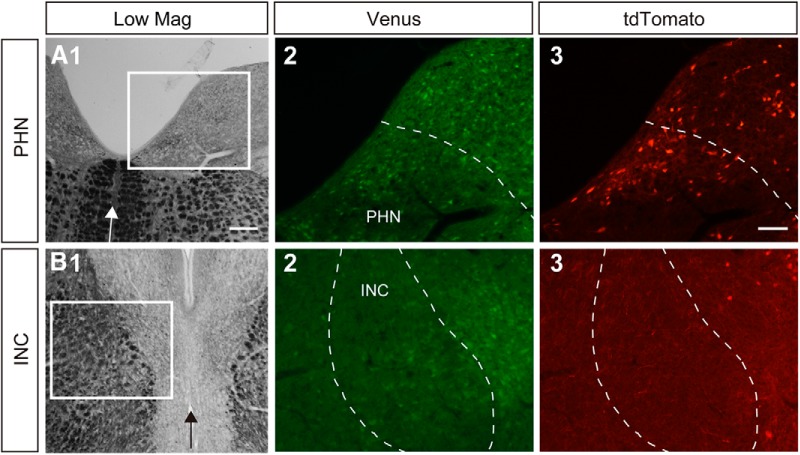
Histologic observation of inhibitory and cholinergic neurons in the INC and the PHN. ***A1***, ***B1***, Low-magnification photomicrographs of frontal sections that included the INC (***A1***) and the PHN (***B1***). Arrow indicates the midline. ***A2***, ***A3***, ***B2***, ***B3***, Fluorescent photomicrographs of the areas outlined by the rectangles in ***A1*** and ***B1***, respectively. The dashed lines indicate the region of the INC (***A2***, ***A3***) and the boundary of the MVN and the PHN (***B2***, ***B3***).

**Figure 3.**
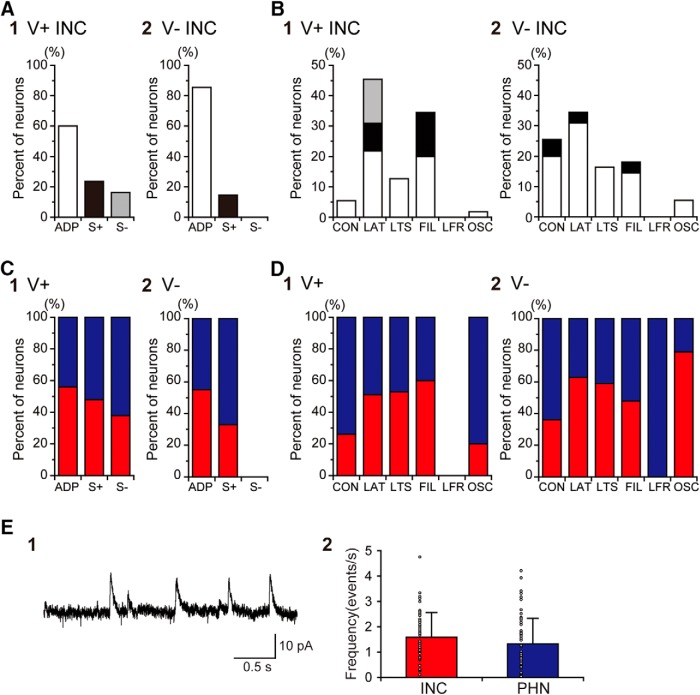
AHP profiles and firing patterns of INC neurons in VGAT-Venus transgenic rats. ***A***, ***B***, Distributions of Venus-expressing (V+ INC; 1) and Venus-nonexpressing (V- INC; 2) neurons classified based on AHP profiles (***A***) and firing patterns (***B***) in the INC. ***C***, ***D***, The proportions of INC (red) and PHN (blue) neurons that exhibited each AHP profile (***C***) or firing pattern (***D***). The percentages of neurons that exhibited each AHP profile or each firing pattern were normalized to the total percentage of neurons exhibiting each property. ***E1***, SMOCs of a V+ INC neuron. ***E2***, Comparison of the frequency of SMOCs between INC and PHN neurons. The plots indicate data obtained from individual neurons, and the bars represent the average value in the INC (red) and PHN (blue) neurons.

In the PHN, cholinergic neurons are present in addition to excitatory glutamatergic neurons and inhibitory GABAergic/glycinergic neurons, which are major neuronal population (McCrea and Horn, 2006). In a previous study using double transgenic rats that were generated by crossing the ChAT-tdTomato and VGAT-Venus transgenic rats, these three types of neurons were identified separately under fluorescence microscopy of brain slices (Saito et al., 2015). In these rats, glutamatergic neurons expressed neither tdTomato nor Venus [double-negative (D-)], cholinergic neurons expressed tdTomato alone, and GABAergic/glycinergic inhibitory neurons expressed Venus alone. The presence of tdTomato-expressing cholinergic neurons and Venus-expressing inhibitory neurons in the PHN was confirmed by observing the fluorescence of the slices obtained from the double transgenic rats (Fig. 2*A*). On the other hand, a previous study reported no observation of ChAT-positive neurons in the INC (Tago et al., 1989). In support of this finding, we found no INC neurons that expressed tdTomato in ChAT-tdTomato transgenic rats (Fig. 2*B3*), although a few tdTomato-expressing neurons were observed near the boundary of the INC. tdTomato-expressing axonal fibers were frequently observed in the INC (Fig. 2*B3*). Electrophysiological studies showed that stimulation of the INC induced excitatory and inhibitory synaptic potentials in motoneurons of the trochlear and oculomotor nuclei and contralateral INC neurons (Schwindt et al., 1974; Nakao and Shiraishi, 1985; Izawa et al., 2007; Sugiuchi et al., 2013). Immunocytochemical and *in situ* hybridization studies showed the presence of glutamate decarboxylase (GAD)-positive neurons in the INC (Giolli et al., 1985; Horn et al., 2003). We observed Venus-expressing neurons that showed medium-sized somata and a scattered distribution in the INC (Fig. 2*B2*), which may be comparable to the distribution of GAD-positive neurons (Giolli et al., 1985; Horn et al., 2003). Because the presence of a substantial number of neurons showing neurotransmitter phenotypes other than glutamatergic and GABAergic has not been reported in the INC, the major neuronal populations in the INC are considered to be excitatory, presumed as glutamatergic neurons and inhibitory GABAergic neurons. Therefore, it might be possible that the different neuronal distributions between the INC and the PHN are partly attributed to the absence of cholinergic neurons in the INC. We next focused on excitatory and inhibitory neurons and compared the neuronal distributions between the INC and the PHN. To compare the electrophysiological properties of the inhibitory and excitatory neurons in the INC to those in the PHN, we reanalyzed the data of V+ and D- neurons in the PHN, which were obtained in a previous study (Saito et al., 2015). Regarding the intrinsic membrane properties of inhibitory neurons in the INC and the PHN, the input capacitance (*p* < 0.0001), the input resistance (*p* < 0.0001), the spike half-width (*p* = 0.0097), the AHP amplitude (*p* = 0.0007), and the CV of the interspike interval (*p* = 0.036) were significantly different between the V+ INC neurons and the V+ PHN neurons (Table 2). With regard to excitatory neurons, the input resistance (*p* = 0.0002), the spike amplitude (*p* < 0.0001), the spike half-width (*p* < 0.0001), the AHP amplitude (*p* = 0.0063), and the CV of the interspike interval (*p* = 0.0003) were significantly different between the V- INC neurons and the D- PHN neurons (Table 2). These results suggest that both excitatory and inhibitory neurons have different intrinsic properties between the INC and the PHN. Figure 3, *A* and *B*, shows the distributions of inhibitory (1) and excitatory (2) neurons classified based on the AHP profiles (A) and the firing patterns (B) in the INC. Neurons that exhibited the ADP profile were predominant both in inhibitory and excitatory neurons and this tendency was significant in excitatory neurons. A majority of inhibitory neurons exhibited either the LAT pattern or the FIL pattern, whereas excitatory neurons appeared not to show a preferred firing pattern. The firing pattern represented in Figure 1*D4* was observed in one V- INC neuron that was confirmed to be within the INC. Figure 3, *C* and *D*, shows the proportion of inhibitory (1) and excitatory (2) neurons that exhibited each AHP profile and each firing pattern in the INC (red) and the PHN (blue). Excitatory neurons exhibiting the S- profile were not seen in the INC or the PHN (Fig. 3*C2*). The percentage of inhibitory neurons that exhibited the S- profile and the percentage of excitatory neurons that exhibited the S+ profile tended to be higher in the PHN than in the INC. None of the inhibitory neurons in the INC and PHN exhibited the LFR pattern (Fig. 3*D1*). The percentage of excitatory and inhibitory neurons that exhibited the CON pattern was higher in the PHN than in the INC. The proportion of INC and PHN neurons that exhibited the OSC pattern was different between inhibitory and excitatory neurons.

**Table 2.** Summary of electrophysiological properties of inhibitory and noninhibitory neurons in the INC and the PHN

	V+ INC	V+ PHN	V- INC	D- PHN
Input capacitance (pF)	56.0 ± 21.7**	31.5 ± 17.6	54.7 ± 23.8*	47.6 ± 32.5
Input resistance (MΩ)	423.1 ± 187.5**	662.9 ± 296.4	358.0 ± 180.1**	572.9 ± 335.3
Spike amplitude (mV)	72.3 ± 7.8	74.6 ± 7.0	69.7 ± 8.4**	76.9 ± 8.9
Spike half-width (ms)	0.398 ± 0.11**	0.441 ± 0.11	0.39 ± 0.13**	0.54 ± 0.14
AHP amplitude (mV)	28.7 ± 6.1**	32.1 ± 4.9	26.4 ± 5.5**	29.1 ± 5.0
Spontaneous firing rate (spikes/s)	10.4 ± 7.2	9.2 ± 4.5	6.1 ± 5.8	7.1 ± 4.3	
CV of the interspike interval (%)	17.2 ± 29.3*	8.8 ± 12.9	16.3 ± 19.5**	8.3 ± 13.5

The analyses were performed on data collected from 70 V+ and 70 V- PHN neurons and 55 V+ and 55 V- INC neurons. The CVs of the interspike interval were analyzed in the neurons that fired spontaneously (52 V+ INC, 68 V+ PHN, 40 V- INC, and 67 D- PHN). Asterisks indicate significant differences between V+ INC and V+ PHN or between V- INC and D- PHN. *p < 0.05; **p < 0.01.

A previous study demonstrated that PHN neurons exhibit spontaneous miniature outward currents (SMOCs; Saito and Yanagawa, 2013) that display slower kinetics than postsynaptic currents and are induced by Ca^2+^-activated K^+^ channels, which are activated through Ca^2+^-induced Ca^2+^ release from the endoplasmic reticulum via ryanodine receptors (Arima et al., 2001; Cui et al., 2004; Klement et al., 2010). Because SMOCs were frequently observed in V+ PHN neurons (Saito and Yanagawa, 2013), we examined whether the SMOCs were also observed in V+ INC neurons. Of 55 V+ INC neurons, 53 exhibited SMOCs (Fig. 3*E1*). No significant difference was seen in the frequency of SMOCs of V+ neurons between the INC and the PHN (*p* = 0.081; Fig. 3*E2*).

### EPSC responses to burst stimulation

**Figure 4.**
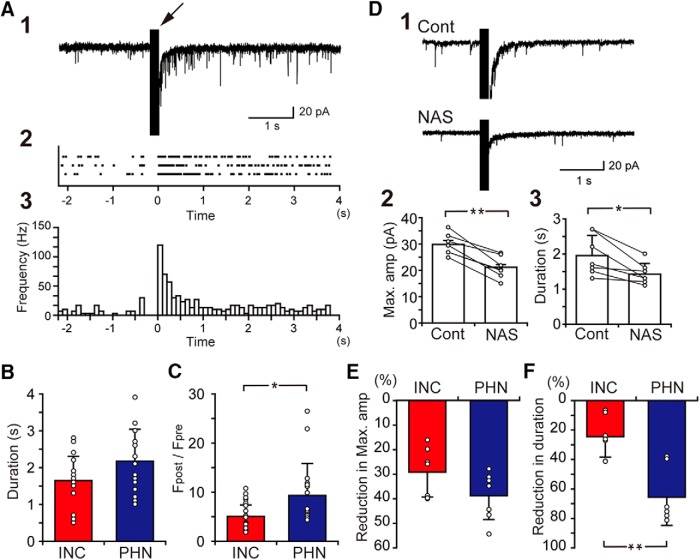
Comparison of EPSC responses to local burst stimulation between INC and PHN neurons. ***A1***, EPSC responses of an INC neuron to burst stimulation (arrow) that was applied in the vicinity of the recorded neuron. ***A2***, Raster plot showing the peak time of each EPSC. The time of the termination of burst stimulation is shown as 0. Each row in the raster represents one recording. ***A3***, A histogram showing the EPSC frequency against time. Bin width of the histograms is 100 ms. ***B***, ***C***, Comparison of the duration of the increased EPSC frequency after the burst stimulation (***B***) and the ratio of the EPSC frequency after the burst stimulation to the frequency before the stimulation (***C***). ***D1***, Spontaneous EPSCs of an INC neuron in a control solution (Cont) and in a solution containing 50 μM NAS (NAS). ***D2***, ***D3***, Comparisons of the amplitude of the maximum EPSC (2) and the duration of the sustained EPSC response (3) between a control solution and a solution containing NAS. ***E***, ***F***, Comparisons of the percentage reduction in the amplitude of the maximum EPSC (***E***) and the reduction in the duration (***F***) caused by NAS between INC and PHN neurons. Plots indicate data obtained from individual neurons, and the bar represents the average value. Asterisks indicate a significant difference between groups (*p < 0.05; **p < 0.01).

We previously demonstrated that under a blockade of inhibitory synaptic transmissions, the application of burst stimulation (100 Hz, 20 pulses) in the vicinity of a recorded neuron within the PHN induced an increase in the frequency of spontaneous EPSCs that lasted for several seconds (Saito and Yanagawa, 2010). This result indicates the presence of local excitatory networks in the PHN, which maintain the increased EPSC frequency. To test whether local excitatory networks also exist in the INC, we investigated spontaneous EPSCs of INC neurons before and after application of burst stimulation. Figure 4*A* shows spontaneous EPSCs of an INC neuron before and after application of burst stimulation (arrow) in the presence of 100 μM picrotoxin and 20 μM strychnine. Spontaneous EPSCs infrequently occurred before the burst stimulation, but the frequency of EPSCs increased after stimulation. The raster plots and the histogram of EPSC frequency constructed for three recording times showed that the high frequency of EPSCs after burst stimulation gradually decreased with time (Fig. 4*A2*,*A3*). The duration of the increased EPSC frequency of this neuron was 1.9 s, which was longer than the duration of the burst stimulation (0.2 s). The EPSC responses were investigated in 17 INC neurons and the mean duration of the increased EPSC frequency was 1.6 ± 0.7 s. When the durations of the INC neurons were compared to those of the previously investigated PHN neurons (*n* = 17, 2.2 ± 0.9 s), there was no significant difference in the duration between the INC neurons and the PHN neurons (*p* = 0.053; Fig. 4*B*). This result suggests the presence of local excitatory networks in the INC, which maintain the increased EPSC frequency. In this experiment, we noticed that the EPSC frequency during 1 s after the burst stimulation was not as high in the INC (26.4 ± 2.0 event/s) compared to in the PHN (31.9 ± 12.0 event/s). The EPSC frequency before the burst stimulation was not significantly different between INC neurons (5.8 ± 3.6 event/s) and PHN neurons (4.2 ± 2.0 event/s, *p* = 0.28). Therefore, the ratio of the EPSC frequency after the burst stimulation to the frequency before the stimulation (F_post_/F_pre_) was compared between the INC and PHN neurons (Fig. 4*C*). The F_post_/F_pre_ of the INC neurons (5.0 ± 2.4) was significantly smaller than that of the PHN neurons (9.3 ± 6.5, *p* = 0.016).

The evidence that the sustained increase in EPSC frequency (sustained EPSC response) was reduced by the application of NAS suggests the involvement of CP-AMPA receptors in the responses (Saito and Yanagawa, 2010). To verify whether CP-AMPA receptors also participate in the sustained EPSC responses in the INC, we investigated the effect of NAS on spontaneous EPSCs of INC neurons (Fig. 4*D*). The application of NAS reduced the incidence of EPSCs (Fig. 4*D1*). Figure 4, *D2* and *D3*, shows comparisons of the maximum EPSC amplitude (2) and the duration of the sustained EPSC responses (3) between the control solution and the solution containing NAS (seven INC neurons were tested). The average amplitude of the maximum EPSC of the INC neurons in the presence of NAS (21.2 ± 4.2 pA) was significantly smaller than that in the control solution (29.9 ± 4.0 pA, *p* = 0.0005; Fig. 4*D2*). The duration in the presence of NAS (1.4 ± 0.3 s) was also significantly shorter than that in the control solution (2.0 ± 0.6 s, *p* = 0.011; Fig. 4*D3*). When the reduction in the maximum EPSC amplitude by NAS was compared between INC and PHN neurons (Fig. 4*E*), the percentage of the reduction in INC neurons (29.2 ± 10.1%) was not significantly different from that in PHN neurons (38.7 ± 9.8%, *p* = 0.098). However, the reduction in the duration by NAS was significantly smaller in the INC (24.5 ± 13.9%) than in the PHN (65.7 ± 18.9%, *p* = 0.0087; Fig. 4*F*).

## Discussion

In this study, we investigated electrophysiological membrane properties of INC neurons and the functional structure of excitatory networks in the INC, both of which were not previously characterized using *in vitro* preparations. Furthermore, we compared the neuronal and network properties of the INC to those of the PHN.

### Neuronal properties of the INC and the PHN

Similar to PHN neurons, a larger population of INC neurons exhibited the ADP profile than other profiles. However, compared with PHN neurons, the proportion of neurons that exhibited the ADP profile was markedly higher in INC neurons. According to studies of PHN neurons and neurons in the medial vestibular nucleus (MVN), the neurons that exhibit biphasic AHP accompanied by ADP are thought to be phasic-tonic neurons that show irregular firing and nonlinear responses to current stimuli (Ris et al., 2001; Beraneck et al., 2003; Idoux et al., 2006; Saito et al., 2008). Indeed, the CV value of the interspike interval, which can be used as an index of discharge regularity, was higher in INC neurons than in PHN neurons (Table 2), suggesting that the number of neurons that show irregular discharge is larger in the INC than in the PHN. These findings indicate that most neurons in the INC may be phasic-tonic neurons. The distribution of neurons based on their firing patterns was also different between the INC and the PHN. A striking feature was that no neuron that exhibited the LFR pattern was found in the INC. Because the LFR pattern was specific to cholinergic neurons in the PHN (Saito et al., 2015), the absence of cholinergic neurons in the INC may be consistent with the absence of neurons that exhibited the LFR pattern. In the PHN, cholinergic neurons that exhibit the LFR pattern show several unique properties: high input capacitance, spontaneous firing at low frequency, overshooting property in response to ramp currents that reflects the active membrane property, hysteretic frequency-current relationship, and expression of GABA_B_ receptors in addition to GABA_A_ and glycine receptors (Zhang et al., 2014; Saito and Yanagawa, 2017). Some cholinergic PHN neurons that exhibit the LFR pattern project into the cerebellar cortex (Zhang et al., 2014). Therefore, the presence or absence of cholinergic effects on other brain areas such as the cerebellum via cholinergic neurons that exhibit the LFR pattern and several unique properties may be a notable difference between the INC and the PHN. However, the different neuronal distribution in the INC and the PHN is not necessarily dependent on the presence of cholinergic neurons in the PHN because the presumed excitatory neurons (V- INC and D- PHN neurons) and inhibitory neurons (V+ INC and V+ PHN neurons) showed different distributions between the INC and the PHN. On the other hand, both INC and PHN neurons exhibited the same AHP profiles and the same firing patterns, except for the LFR pattern. Therefore, the neuronal types in terms of membrane properties are common in the INC and the PHN, but their distributions are different between them. As described above, the proportion of neurons that exhibit phasic and nonlinear properties is inferred to be higher in the INC than in the PHN. This perspective may be supported by the higher percentage of neurons that exhibit the LTS pattern and the lower percentage of neurons that exhibit the CON pattern in the INC than in the PHN. The LTS pattern is mainly caused by T-type Ca^2+^ currents and often observed in neurons that show nonlinear burst discharge (Jahnsen and Llinás, 1984; McCormick et al., 1985; Serafin et al., 1991; for reviews, see Llinás, 1988; McCormick, 2014), whereas the CON pattern shows linear firing responses to various current stimulations (Saito and Yanagawa, 2017).

Preceding *in vitro* studies have shown that MVN neurons are classified into discrete types based on their spike shapes: a type that exhibits monophasic AHP (type A), a type that exhibits biphasic AHP (type B), and an intermediate type (type C; Serafin et al., 1991; Johnston et al., 1994; Beraneck et al., 2003; Camp et al., 2006). Based on previous MVN studies investigating the ionic mechanisms of each AHP profile, the fast component of the AHP is caused by A-type K^+^ currents and/or large conductance-type Ca^2+^-dependent K^+^ currents, whereas the slow component of the AHP is caused mainly by small conductance-type Ca^2+^-dependent K^+^ currents (Serafin et al., 1991; Johnston et al., 1994; Smith et al., 2002; Saito et al., 2008). The ADP is believed to be caused by Ca^2+^-dependent nonselective currents (Haj-Dahmane and Andrade, 1997). In addition to the AHP profiles, our previous studies provided an alternative classification of MVN neurons based on firing pattern (Takazawa et al., 2004; Saito and Ozawa, 2007). The firing patterns of MVN neurons are mostly similar to those of PHN neurons except for the OSC pattern that has not been observed in MVN neurons (Takazawa et al., 2004; Zhang et al., 2014). Therefore, the CON, LAT, LTS, and FIL patterns are common firing pattern to PHN, INC, and MVN neurons. In our previous studies, a plateau-like potential and a large phase shift were not observed in most PHN neurons (Shino et al., 2008, 2011; Saito et al., 2015; Saito and Yanagawa, 2017), suggesting that the signals directing gaze holding are not generated solely by the intrinsic properties of the individual neurons in the neural integrator. Therefore, the network properties are essential to generate gaze holding signals, and how individual neurons are embedded in the integrator networks is important. Regarding the networks in the PHN, cascade-like connections from velocity-coding phasic neurons to position-coding tonic neurons have been proposed (Escudero et al., 1992; Delgado-García et al., 2006). Based on the firing patterns, neurons that exhibit the CON, LAT, and FIL patterns fire tonically, whereas neurons that exhibit the LTS pattern, which corresponds to the firing pattern of B + LTS MVN neurons (Serafin et al., 1991; Sekirnjak and du Lac, 2002; Camp et al., 2010), show phasic activity. Therefore, when the neurons are embedded in the cascade-like connections, the signals from the premotor areas reach the LTS type and are transmitted to the CON, LAT, and FIL types. Neurons that exhibit the OSC pattern were observed in the PHN and INC but not in the MVN. This neuron type is similar to type D neurons reported by Idoux et al. (2006), with respect to the oscillatory firing (Saito et al., 2015). Type D neurons show bistable plateau-like responses through an NMDA-dependent mechanism, which may be used for the persistency of neural activities in the integrator network. Therefore, the OSC type may play an instrumental role in the integrator networks in the PHN and the INC.

### Excitatory network properties of the INC and the PHN

Gaze holding is supported by tonic activity that is produced via the neural integrator (Robinson, 1975, 1989; Fukushima et al., 1992; Fukushima and Kaneko, 1995; Moschovakis, 1997; Leigh and Zee, 2015). Experimental findings suggest that positive feedback circuits through contralateral integrator regions and cerebellar circuits (Aksay et al., 2001, 2003, 2007; Leigh and Zee, 2015) and the sustained depolarization of PHN neurons mediated by cholinergic inputs (Navarro-López Jde et al., 2004, 2005) participate in tonic activity. In addition, a previous study suggests the contribution of the sustained activation of local excitatory networks to tonic activity (Saito and Yanagawa, 2010). In the present study, analysis of spontaneous EPSCs of INC neurons revealed that the frequency of EPSCs after burst stimulation increased for a few seconds. This enhancement in EPSC frequency following burst stimulation is similar to the sustained EPSC response that was observed in PHN neurons (Saito and Yanagawa, 2010), suggesting that local excitatory networks that activate the sustained EPSC response also exist in the INC. However, the fact that the increase in EPSC frequency after the burst stimulation was not as drastic in the INC compared to the PHN raises a possibility that the number of neurons that participates in excitatory networks and/or the degree of the excitatory connections in the INC may be smaller than those in the PHN. It has been shown that both the PHN and the INC show reciprocal connections with the vestibular nuclei (Fukushima, 1987; McCrea and Horn, 2006; Leigh and Zee, 2015). Therefore, part of the MVN adjacent to the PHN also participates in the horizontal integrator (Cannon and Robinson, 1987; McFarland and Fuchs, 1992; Sylvestre et al., 2003). A study in the monkey showed that transection of the medial longitudinal fasciculus, which includes the fibers participating in connections between the vestibular nuclei and the INC, results in an impairment of gaze holding after a vertical saccade (Evinger et al., 1977). These findings suggest that the networks to the vestibular nuclei are necessary for the neural integrator to sufficiently function. The moderate increase in the EPSC frequency of INC neurons after the burst stimulation may be attributed to the slice preparations, as the networks between the INC and the vestibular nuclei are disconnected. Therefore, activation of the reverberating circuits between the INC and the vestibular nuclei may be necessary for activating the excitatory networks of the neural integrator for vertical gaze holding. On the other hand, sustained EPSC responses and a drastic increase in EPSC frequency were observed even when the PHN was isolated from other brainstem regions by dissection (Saito and Yanagawa, 2010). This finding suggests that the excitatory networks of the neural integrator for horizontal gaze holding can be activated by recurrent networks within the PHN.

A previous study showed that activation of excitatory networks in the PHN are mediated by CP-AMPA receptors (Saito and Yanagawa, 2010). The burst inputs proportional to velocity signals from premotor burst neurons cause cumulative increases in intracellular Ca^2+^ concentrations of PHN neurons by repetitive activation of CP-AMPA receptors. The increase in the Ca^2+^ may activate calcium-dependent nonselective cation (CAN) channels, which show slow deactivation kinetics (Morisset and Nagy, 1999; Di Prisco et al., 2000; Fransén et al., 2006). CAN channel-dependent sustained depolarization may persistently activate PHN networks (Saito and Yanagawa, 2010). However, this scenario may not be necessarily applicable to INC networks, because the contribution of CP-AMPA receptors on the sustained EPSC responses was not as great in INC neurons as in PHN neurons (Fig. 4*F*). Although we did not test other mechanisms regarding the sustained EPSC responses in the INC, the present findings imply different mechanisms for the generation of sustained activities between the PHN and the INC. In this study, we found different neuronal distributions and different properties of excitatory networks between the PHN and the INC. Because the PHN and the INC show reciprocal connections (McCrea and Horn, 2006; Leigh and Zee, 2015), clarifying how these nuclei interact is necessary for determining the network mechanisms of these integrators.

## Synthesis

Reviewing Editor: Kate Jeffery, University College London Decisions are customarily a result of the Reviewing Editor and the peer reviewers coming together and discussing their recommendations until a consensus is reached. When revisions are invited, a fact-based synthesis statement explaining their decision and outlining what is needed to prepare a revision will be listed below. The following reviewer(s) agreed to reveal their identity: Aaron Camp, Mathieu Beraneck

- Although the authors have clarified the distinctions between both LTS firing patterns (Fig 1C 3 and 4) in text, could the authors please adjust the labelling so to highlight this difference? Perhaps changing the name of 4 to LTS subtype?

- Could the authors in Figure 1A change the colour of the font from black to white for better contrast and easier legibility.

-Line 467, suggest to delete "more"

-Line 479: suggest "persistency" instead of "robustness".

-Line 480: suggest "the OSC type may play an instrumental role in the integrator networks of both the PHN and INC"

-Line 484: suggest "supported" instead of "attained"

-Line 867: suggest to put (4) between (3) and (5) for ease of reading.

## Author Response

Manuscript number: eN-NWR-0180-17R1

Reply to the comments from the reviewers:

Synthesis of Reviews:

Computational Neuroscience Model Code Accessibility Comments for Author (Required):

N/A

Significance Statement Comments for Author (Required):

N/A

Comments on the Visual Abstract for Author (Required):

The visual abstract is insufficiently informative - it needs to be understandable by a non-specialist who has not yet read the paper, and it thus cannot have a lot of acronyms and jargon. it is advisable to have the abstract reviewed by a colleague from a different field, to check for understandability.

Reply:

We have modified the Visual Abstract according to the opinion of a colleague who studies in a different field. We believe that this abstract is mostly understandable even by a non-specialist.

Synthesis Statement for Author (Required):

- Although the authors have clarified the distinctions between both LTS firing patterns (Fig 1C 3 and 4) in text, could the authors please adjust the labelling so to highlight this difference? Perhaps changing the name of 4 to LTS subtype?

Reply:

We appreciate the reviewer's suggestion. We have changed the label of 4 to LTS subtype (Fig. 1).

- Could the authors in Figure 1A change the colour of the font from black to white for better contrast and easier legibility.

Reply:

We have changed the color of the broken line and the letters from black to white (Fig. 1).

-Line 467, suggest to delete “more”

Reply:

We have deleted the word (Line 467; the page and line numbers will be changed from the original file in the upload process. Therefore, we indicate the line number in the previously uploaded file).

-Line 479: suggest “persistency” instead of “robustness”.

Reply:

We have changed the word (Line 479).

-Line 480: suggest “the OSC type may play an instrumental role in the integrator networks of both the PHN and INC”

Reply:

We agree with the suggestion. We have revised the description (Line 480).

-Line 484: suggest “supported” instead of “attained”

Reply:

We have changed the word (Line 484).

-Line 867: suggest to put (4) between (3) and (5) for ease of reading.

Reply:

We have changed the description the type of (4) as a LTS subtype. We have modified the descriptions as the reviewer suggested (Line 867).

## References

[B1] Aksay E, Gamkrelidze G, Seung HS, Baker R, Tank DW (2001) *In vivo* intracellular recording and perturbation of persistent activity in a neural integrator. Nat Neurosci 4:184–193. 10.1038/84023 11175880

[B2] Aksay E, Baker R, Seung HS, Tank DW (2003) Correlated discharge among cell pairs within the oculomotor horizontal velocity-to-position integrator. J Neurosci 23:10852–10858. 14645478 10.1523/JNEUROSCI.23-34-10852.2003 PMC6740981

[B3] Aksay E, Olasagasti I, Mensh BD, Baker R, Goldman MS, Tank DW (2007) Functional dissection of circuitry in a neural integrator. Nat Neurosci 10:494–504. 10.1038/nn1877 17369822 PMC2803116

[B4] Arima J, Matsumoto N, Kishimoto K, Akaike N (2001) Spontaneous miniature outward currents in mechanically dissociated rat Meynert neurons. J Physiol 534:99–107. 11432995 10.1111/j.1469-7793.2001.00099.x PMC2278683

[B5] Arnold DB, Robinson DA (1997) The oculomotor integrator: testing of a neural network model. Exp Brain.Res 113:57–74. 9028775 10.1007/BF02454142

[B6] Beraneck M, Hachemaoui M, Idoux E, Ris L, Uno A, Godaux E, Vidal PP, Moore LE, Vibert N (2003) Long-term plasticity of ipsilesional medial vestibular nucleus neurons after unilateral labyrinthectomy. J Neurophysiol 90:184–203. 10.1152/jn.01140.2002 12649317

[B7] Bobker DH, Williams JT (1990) Serotonin-mediated inhibitory postsynaptic potential in guinea-pig prepositus hypoglossi and feedback inhibition by serotonin. J Physiol 422:447–462. 2141079 10.1113/jphysiol.1990.sp017994 PMC1190142

[B8] Bobker DH, Williams JT (1995) The serotonergic inhibitory postsynaptic potential in prepositus hypoglossi is mediated by two potassium currents. J Neurosci 15:223–229. 7529825 10.1523/JNEUROSCI.15-01-00223.1995 PMC6578297

[B9] Büttner U, Büttner-Ennever JA (2006) Present concepts of oculomotor organization. Prog Brain Res 151:1–42. 10.1016/S0079-6123(05)51001-X 16221584

[B10] Camp AJ, Callister RJ, Brichta AM (2006) Inhibitory synaptic transmission differs in mouse type A and B medial vestibular nucleus neurons in vitro. J Neurophysiol 95:3208–3218. 10.1152/jn.01001.2005 16407430

[B11] Camp AJ, Lim R, Anderson WB, Schofield PR, Callister RJ, Brichta AM (2010) Attenuated glycine receptor function reduces excitability of mouse medial vestibular nucleus neurons. Neuroscience 170:348–360. 10.1016/j.neuroscience.2010.06.040 20600650

[B12] Cannon SC, Robinson DA (1985) An improved neural-network model for the neural integrator of the oculomotor system: more realistic neuron behavior. Biol Cybern 53:93–108. 4084616 10.1007/BF00337026

[B13] Cannon SC, Robinson DA (1987) Loss of the neural integrator of the oculomotor system from brain stem lesions in monkey. J Neurophysiol 57:1383–1409. 3585473 10.1152/jn.1987.57.5.1383

[B14] Cannon SC, Robinson DA, Shamma S (1983) A proposed neural network for the integrator of the oculomotor system. Biol Cybern 49:127–136. 6661444 10.1007/BF00320393

[B15] Chimoto S, Iwamoto Y, Yoshida K (1999) Projections and firing properties of down eye-movement neurons in the interstitial nucleus of Cajal in the cat. J Neurophysiol 81:1199–1211. 10085347 10.1152/jn.1999.81.3.1199

[B16] Crawford JD, Cadera W, Vilis T (1991) Generation of torsional and vertical eye position signals by the interstitial nucleus of Cajal. Science 252:1551–1553. 2047862 10.1126/science.2047862

[B17] Cui G, Okamoto T, Morikawa H (2004) Spontaneous opening of T-type Ca2+ channels contributes to the irregular firing of dopamine neurons in neonatal rats. J Neurosci 24:11079–11087. 10.1523/JNEUROSCI.2713-04.2004 15590924 PMC1454359

[B18] Delgado-García JM, Vidal PP, Gómez C, Berthoz A (1989) A neurophysiological study of prepositus hypoglossi neurons projecting to oculomotor and preoculomotor nuclei in the alert cat. Neuroscience 29:291–307. 2725860 10.1016/0306-4522(89)90058-4

[B19] Delgado-García JM, Yajeya J, Navarro-López Jde D (2006) A cholinergic mechanism underlies persistent neural activity necessary for eye fixation. Prog Brain Res 154:211–224. 10.1016/S0079-6123(06)54011-7 17010712

[B20] Draye JP, Cheron G, Libert G, Godaux E (1997) Emergence of clusters in the hidden layer of a dynamic recurrent neural network. Biol Cybern 76:365–374. 10.1007/s004220050350 9237362

[B21] Escudero M, de la Cruz RR, Delgado-García JM (1992) A physiological study of vestibular and prepositus hypoglossi neurons projecting to the abducens nucleus in the alert cat. J Physiol 458:539–560. 1302278 10.1113/jphysiol.1992.sp019433 PMC1175171

[B22] Evinger LC, Fuchs AF, Baker R (1977) Bilateral lesions of the medial longitudinal fasciculus in monkeys: effects on the horizontal and vertical components of voluntary and vestibular induced eye movements. Exp Brain Res 28:1–20. 407093 10.1007/BF00237082

[B23] Faul F, Erdfelder E, Lang AG, Buchner A (2007) G*Power 3: a flexible statistical power analysis program for the social, behavioral, and biomedical sciences. Behav Res Methods 39:175–191. 17695343 10.3758/bf03193146

[B24] Fransén E, Tahvildari B, Egorov AV, Hasselmo ME, Alonso AA (2006) Mechanism of graded persistent cellular activity of entorhinal cortex layer V neurons. Neuron 49:735–746. 10.1016/j.neuron.2006.01.036 16504948

[B25] Fukushima K (1987) The interstitial nucleus of Cajal and its role in the control of movements of head and eyes. Prog Neurobiol 29:107–192. 3108957 10.1016/0301-0082(87)90016-5

[B26] Fukushima K (1991) The interstitial nucleus of Cajal in the midbrain reticular formation and vertical eye movement. Neurosci Res 10:159–187. 1650435 10.1016/0168-0102(91)90055-4

[B27] Fukushima K, Kaneko CR (1995) Vestibular integrators in the oculomotor system. Neurosci Res 22:249–259. 7478288 10.1016/0168-0102(95)00904-8

[B28] Fukushima K, Harada C, Fukushima J, Suzuki Y (1990a) Spatial properties of vertical eye movement-related neurons in the region of the interstitial nucleus of Cajal in awake cats. Exp Brain Res 79:25–42. 2311702 10.1007/BF00228871

[B29] Fukushima K, Fukushima J, Harada C, Ohashi T, Kase M (1990b) Neuronal activity related to vertical eye movement in the region of the interstitial nucleus of Cajal in alert cats. Exp Brain Res 79:43–64. 2311703 10.1007/BF00228872

[B30] Fukushima K, Kaneko CR, Fuchs AF (1992) The neuronal substrate of integration in the oculomotor system. Prog Neurobiol 39:609–639. 1410443 10.1016/0301-0082(92)90016-8

[B31] Galiana HL, Outerbridge JS (1984) A bilateral model for central neural pathways in vestibuloocular reflex. J Neurophysiol 51:210–241. 6608579 10.1152/jn.1984.51.2.210

[B32] Giolli RA, Peterson GM, Ribak CE, McDonald HM, Blanks RH, Fallon JH (1985) GABAergic neurons comprise a major cell type in rodent visual relay nuclei: an immunocytochemical study of pretectal and accessory optic nuclei. Exp Brain Res 61:194–203. 10.1007/BF00235635 3002835

[B33] Haj-Dahmane S, Andrade R (1997) Calcium-activated cation nonselective current contributes to the fast afterdepolarization in rat prefrontal cortex neurons. J Neurophysiol 78:1983–1989. 9325366 10.1152/jn.1997.78.4.1983

[B34] Helmchen C, Rambold H, Fuhry L, Büttner U (1998) Deficits in vertical and torsional eye movements after uni- and bilateral muscimol inactivation of the interstitial nucleus of Cajal of the alert monkey. Exp Brain Res 119:436–452. 9588778 10.1007/s002210050359

[B35] Horn AK, Helmchen C, Wahle P (2003) GABAergic neurons in the rostral mesencephalon of the macaque monkey that control vertical eye movements. Ann NY Acad Sci 1004:19–28. 14662444 10.1196/annals.1303.003

[B36] Idoux E, Serafin M, Fort P, Vidal PP, Beraneck M, Vibert N, Mühlethaler M, Moore LE (2006) Oscillatory and intrinsic membrane properties of guinea pig nucleus prepositus hypoglossi neurons in vitro. J Neurophysiol 96:175–196. 10.1152/jn.01355.2005 16598060

[B37] Idoux E, Eugène D, Chambaz A, Magnani C, White JA, Moore LE (2008) Control of neuronal persistent activity by voltage-dependent dendritic properties. J Neurophysiol 100:1278–1286. 10.1152/jn.90559.2008 18632879 PMC2544453

[B38] Izawa Y, Sugiuchi Y, Shinoda Y (2007) Neural organization of the pathways from the superior colliculus to trochlear motoneurons. J Neurophysiol 97:3696–3712. 10.1152/jn.01073.2006 17488977

[B39] Jahnsen H, Llinás R (1984) Electrophysiological properties of guinea-pig thalamic neurons: an *in vitro* study. J Physiol 349:205–226. 6737292 10.1113/jphysiol.1984.sp015153 PMC1199334

[B40] Johnston AR, MacLeod NK, Dutia MB (1994) Ionic conductances contributing to spike repolarization and after-potentials in rat medial vestibular nucleus neurones. J Physiol 481:61–77. 10.1113/jphysiol.1994.sp020419 7531769 PMC1155866

[B41] King WM, Fuchs AF, Magnin M (1981) Vertical eye movement-related responses of neurons in midbrain near intestinal nucleus of Cajal. J Neurophysiol 46:549–562. 7299433 10.1152/jn.1981.46.3.549

[B42] Klement G, Druzin M, Haage D, Malinina E, Arhem P, Johansson S (2010) Spontaneous ryanodine-receptor-dependent Ca2+-activated K+ currents and hyperpolarizations in rat medial preoptic neurons. J Neurophysiol 103:2900–2911. 10.1152/jn.00566.2009 20457857

[B43] Kolkman KE, Moghadam SH, du Lac S (2011) Intrinsic physiology of identified neurons in the prepositus hypoglossi and medial vestibular nuclei. J Vestib Res 21:33–47. 10.3233/VES-2011-0394 21422541 PMC3285271

[B44] Lee MM, Arrenberg AB, Aksay ER (2015) A structural and genotypic scaffold underlying temporal integration. J Neurosci 35:7903–7920. 10.1523/JNEUROSCI.3045-14.2015 25995475 PMC4438132

[B45] Leigh RJ, Zee DS (2015) The neurology of eye movements. Oxford: Oxford University Press.

[B46] Llinás R (1988) The intrinsic electrophysiological properties of mammalian neurons: insights into central nervous system function. Science 242:1654–1664. 3059497 10.1126/science.3059497

[B47] Lopez-Barneo J, Darlot C, Berthoz A, Baker R (1982) Neuronal activity in prepositus nucleus correlated with eye movement in the alert cat. J Neurophysiol 47:329–352. 7062103 10.1152/jn.1982.47.2.329

[B48] Major G, Tank D (2004) Persistent neural activity: prevalence and mechanisms. Curr Opin Neurobiol 14:675–684. 10.1016/j.conb.2004.10.017 15582368

[B49] McCormick DA (2014) Membrane potential and action potential. In: From molecules to networks: an introduction to cellular and molecular neuroscience, Ed 3 ( Byrne JH, Heidelberger R, Waxham MN eds), pp 351–376. San Diego: Academic.

[B50] McCormick DA, Connors BW, Lighthall JW, Prince DA (1985) Comparative electrophysiology of pyramidal and sparsely spiny stellate neurons of the cortex. J Neurophysiol 54:782–806. 2999347 10.1152/jn.1985.54.4.782

[B51] McCrea RA, Horn AK (2006) Nucleus prepositus. Prog Brain Res 151:205–230. 10.1016/S0079-6123(05)51007-0 16221590

[B52] McFarland JL, Fuchs AF (1992) Discharge patterns in nucleus prepositus hypoglossi and adjacent medial vestibular nucleus during horizontal eye movement in behaving macaques. J Neurophysiol 68:319–332. 1517825 10.1152/jn.1992.68.1.319

[B53] Miller NR (1985) The neural control of eye movements. In: Walsh and Hoyt's clinical neuro-ophthalmology, Vol 2, Ed 4 Baltimore: Williams & Wilkins.

[B54] Morisset V, Nagy F (1999) Ionic basis for plateau potentials in deep dorsal horn neurons of the rat spinal cord. J Neurosci 19:7309–7316. 10460237 10.1523/JNEUROSCI.19-17-07309.1999 PMC6782528

[B55] Moschovakis AK (1997) The neural integrators of the mammalian saccadic system. Front Biosci 2:D552–D577. 9341239 10.2741/a212

[B56] Nagai T, Ibata K, Park ES, Kubota M, Mikoshiba K, Miyawaki A (2002) A variant of yellow fluorescent protein with fast and efficient maturation for cell-biological applications. Nat Biotechnol 20:87–90. 10.1038/nbt0102-87 11753368

[B57] Nakao S, Shiraishi Y (1985) Direct excitatory and inhibitory synaptic inputs from the medial mesodiencephalic junction to motoneurons innervating extraocular oblique muscles in the cat. Exp Brain Res 61:62–72. 3002837 10.1007/BF00235621

[B58] Navarro-López Jde D, Alvarado JC, Márquez-Ruiz J, Escudero M, Delgado-García JM, Yajeya J (2004) A cholinergic synaptically triggered event participates in the generation of persistent activity necessary for eye fixation. J Neurosci 24:5109–5118. 10.1523/JNEUROSCI.0235-04.2004 15175380 PMC6729203

[B59] Navarro-López Jde D, Delgado-García JM, Yajeya J (2005) Cooperative glutamatergic and cholinergic mechanisms generate short-term modifications of synaptic effectiveness in prepositus hypoglossi neurons. J Neurosci 25:9902–9906. 10.1523/JNEUROSCI.2061-05.2005 16251437 PMC6725563

[B60] Paxinos G, Watson C (2007) The rat brain in stereotaxic coordinates, Ed 6 San Diego: Academic Press.

[B61] Ris L, Hachemaoui M, Vibert N, Godaux E, Vidal PP, Moore LE (2001) Resonance of spike discharge modulation in neurons of the guinea pig medial vestibular nucleus. J Neurophysiol 86:703–716. 11495944 10.1152/jn.2001.86.2.703

[B62] Robinson DA (1975) Oculomotor control signals. In: Basic mechanisms of ocular motility and their clinical implications ( Lennerstrand G, Bach-y-Rita P, eds), pp 337–374. Oxford: Pergamon.

[B63] Robinson DA (1989) Integrating with neurons. Ann Rev Neurosci 12:33–45. 10.1146/annurev.ne.12.030189.000341 2648952

[B64] Rutherford JG, Gwyn DG (1982) A light and electron microscopic study of the interstitial nucleus of Cajal in rat. J Comp Neur 205:327–340. 10.1002/cne.902050403 7096624

[B65] Saito Y, Ozawa S (2007) Membrane properties of rat medial vestibular nucleus neurons *in vivo* . Neurosci Res 59:215–223. 10.1016/j.neures.2007.06.1479 17720270

[B66] Saito Y, Yanagawa Y (2010) Synaptic mechanism for the sustained activation of oculomotor integrator circuits in the rat prepositus hypoglossi nucleus: contribution of Ca^2+^-permeable AMPA receptors. J Neurosci 30:15735–15746. 10.1523/JNEUROSCI.2814-10.2010 21106813 PMC6633753

[B67] Saito Y, Yanagawa Y (2013) Ca^2+^-activated ion currents triggered by ryanodine receptor-mediated Ca^2+^ release control the firing of inhibitory neurons in the prepositus hypoglossi nucleus. J Neurophysiol 109:389–404. 10.1152/jn.00617.2012 23100137

[B68] Saito Y, Yanagawa Y (2017) Distinct response properties of rat prepositus hypoglossi nucleus neurons classified on the basis of firing patterns. Neurosci Res 121:18–28. 10.1016/j.neures.2017.03.003 28288866

[B69] Saito Y, Takazawa T, Ozawa S (2008) Relationship between afterhyperpolarization profiles and the regularity of spontaneous firings in rat medial vestibular nucleus neurons. Eur J Neurosci 28:288–298. 10.1111/j.1460-9568.2008.06338.x 18702700

[B70] Saito Y, Shino M, Yanagawa Y (2012) Characterization of ionic channels underlying the specific firing pattern of a novel neuronal subtype in the rat prepositus hypoglossi nucleus. Neurosci Res 73:32–41. 10.1016/j.neures.2012.02.011 22401839

[B71] Saito Y, Zhang Y, Yanagawa Y (2015) Electrophysiological and morphological properties of neurons in the prepositus hypoglossi nucleus that express both ChAT and VGAT in a double-transgenic rat model. Eur J Neurosci 41:1036–1048. 10.1111/ejn.12878 25808645

[B72] Schwindt PC, Precht W, Richter A (1974) Monosynaptic excitatory and inhibitory pathways from medial midbrain nuclei to trochlear motoneurons. Exp Brain Res 20:223–238. 4426351 10.1007/BF00238314

[B73] Sekirnjak C, du Lac S (2002) Intrinsic firing dynamics of vestibular nucleus neurons. J Neurosci 22:2083–2095. 11896148 10.1523/JNEUROSCI.22-06-02083.2002 PMC6758259

[B74] Serafin M, de Waele C, Khateb A, Vidal PP, Mühlethaler M (1991) Medial vestibular nucleus in the guinea-pig. II. Ionic basis of the intrinsic membrane properties in brainstem slices. Exp Brain Res 84:426–433. 1648506 10.1007/BF00231465

[B75] Seung HS, Lee DD, Reis BY, Tank DW (2000) Stability of the memory of eye position in a recurrent network of conductance-based model neurons. Neuron 26:259–271. 10798409 10.1016/s0896-6273(00)81155-1

[B76] Shino M, Ozawa S, Furuya N, Saito Y (2008) Membrane properties of excitatory and inhibitory neurons in the rat prepositus hypoglossi nucleus. Eur J Neurosci 27:2413–2424. 10.1111/j.1460-9568.2008.06204.x 18445229

[B77] Shino M, Kaneko R, Yanagawa Y, Kawaguchi Y, Saito Y (2011) Electrophysiological characteristics of inhibitory neurons of the prepositus hypoglossi nucleus as analyzed in Venus-expressing transgenic rats. Neuroscience 197:89–98. 10.1016/j.neuroscience.2011.09.017 21952130

[B78] Smith MR, Nelson AB, DU Lac S (2002) Regulation of firing response gain by calcium-dependent mechanisms in vestibular nucleus neurons. J Neurophysiol 87:2031–2042. 10.1152/jn.00821.2001 11929921

[B79] Sugiuchi Y, Takahashi M, Shinoda Y (2013) Input-output organization of inhibitory neurons in the interstitial nucleus of Cajal projecting to the contralateral trochlear and oculomotor nucleus. J Neurophysiol 110:640–657. 10.1152/jn.01045.2012 23657283

[B80] Sylvestre PA, Choi JT, Cullen KE (2003) Discharge dynamics of oculomotor neural integrator neurons during conjugate and disjunctive saccades and fixation. J Neurophysiol 90:739–754. 10.1152/jn.00123.2003 12672779

[B81] Tago H, McGeer PL, McGeer EG, Akiyama H, Hersh LB (1989) Distribution of choline acetyltransferase immunopositive structures in the rat brainstem. Brain Res 495:271–297. 10.1016/0006-8993(89)90221-7 2765931

[B82] Takazawa T, Saito Y, Tsuzuki K, Ozawa S (2004) Classification of neuron types based on molecular, electrophysiological, and morphological characteristics in the rat medial vestibular nucleus. J Neurophysiol 92:3106–3120. 10.1152/jn.00494.2004 15240763

[B83] Uematsu M, Hirai H, Karube F, Ebihara S, Kato M, Abe K, Obata K, Yoshida S, Hirabayashi M, Yanagawa Y, Kawaguchi Y (2008) Quantitative chemical composition of cortical GABAergic neurons revealed in transgenic venus-expressing rats. Cereb Cortex 18:315–330. 10.1093/cercor/bhm056 17517679

[B84] Di Prisco GV, Pearlstein E, Le Ray D, Robitaille R, Dubuc R (2000) A cellular mechanism for the transformation of a sensory input into a motor command. J Neurosci 20:8169–8176. 11050140 10.1523/JNEUROSCI.20-21-08169.2000 PMC6772722

[B85] Zhang Y, Kaneko R, Yanagawa Y, Saito Y (2014) The vestibulo- and preposito-cerebellar cholinergic neurons of a ChAT-tdTomato transgenic rat exhibit heterogeneous firing properties and the expression of various neurotransmitter receptors. Eur J Neurosci 39:1294–1313. 10.1111/ejn.12509 24593297

[B86] Zhang Y, Yanagawa Y, Saito Y (2016) Nicotinic acetylcholine receptor-mediated responses in medial vestibular and prepositus hypoglossi nuclei neurons showing distinct neurotransmitter phenotypes. J Neurophysiol 115:2649–2657. 10.1152/jn.00852.2015 26936981 PMC4922479

